# Structure and Properties of Zr-Based Bulk Metallic Glasses in As-Cast State and After Laser Welding

**DOI:** 10.3390/ma11071117

**Published:** 2018-06-29

**Authors:** Wirginia Pilarczyk

**Affiliations:** Faculty of Mechanical Engineering, Silesian University of Technology, Konarskiego 18a St., 44-100 Gliwice, Poland; wirginia.pilarczyk@polsl.pl; Tel.: +48-32-237-20-21

**Keywords:** bulk metallic glasses, welding, laser beam welding, AFM, HRTEM

## Abstract

Laser-beam fusion welding processes enable the increase of the size of metallic glass parts, and therefore facilitate the application of this new material in different products. To get exceptional properties of the material after the welding process, characteristics of the weld structure in the heat affected zone and in the fusion zone should be predicted. The main goal of this work is to study the structure and specific characteristics of the Zr_55_Cu_30_Ni_5_Al_10_ alloy after the casting process and after the laser welding process. Electron microscopy observation confirmed that the amorphous phase was present in the fusion zone and amorphous–crystalline phase was present in the heat-affected zone. Higher nanohardness and reduced Young’s modulus values were demonstrated for laser welds using higher impulse energy (2.78 J) and impulse peak power (1000 W).

## 1. Introduction

### 1.1. Current State-of-the-Art Technologies Used in the Joining of Bulk Metallic Glasses

Recent years have seen the intense development of bulk metallic glasses (BMGs). Presently, maximum dimensions of manufactured parts reach diameters of tens of millimeters, yet the thickness of homogenous metallic glass is still overly thin for BMGs to find industrial applications. Sufficient dimensions and the appropriately high thermal stability of specimens are required for BMGs to be used in selected engineering processes. The development of methods enabling the joining of BMGs is the precondition of their industrial application [1,2,3].

Generally, in some specific cases the properties of BMGs are diversified and better than the properties of their equivalents having the crystalline structure. Due to a chemical uniformity of their structure BMGs are characterized by a high strength and corrosion resistance. The values of their Young’s modulus are similar to those of their crystalline equivalents, yet they do not show the anisotropy of elastic properties and cannot accommodate a plastic strain. Amorphous materials have a lower electrical conductance than the crystalline alloys with the same chemical composition. For this reason, BMGs can be applied as functional materials and materials having favorable (desired) characteristic structures [4,5,6,7,8,9].

The making of small-sized specimens is connected, among other things, with the limited glass forming ability (GFA) of test alloys. For this reason, numerous research works are concerned with various methods enabling not only the production, but also the joining of BMGs.

Until today, it has been possible to join BMGs having various chemical compositions (e.g., Pd_40_Ni_40_P_20_, Pd_40_Cu_30_Ni_10_P_20_, Zr_55_Al_10_Ni_5_Cu_30_, and Zr_41_Ti_14_Cu_12_Ni_10_Be_23_) using welding processes in the supercooled liquid phase and using welding methods with the liquid phase content [9,10]. Presently, laser welding, viewed as a modern joining method, arouses interest among scientists because of the possibilities resulting from the use of the laser beam, particularly in terms of laser beam features including obtainable weld penetration depths and high-energy density [9]. Laser welding is characterized by high cohesion in space and time as well as by a narrow wavelength range. The primary laser radiation features include monochromaticity, the minimum divergence of the laser beam, parallelism, and high radiation energy [11,12,13].

The analysis of related reference publications revealed that tests involving the joining of BMGs performed using the laser beam were undertaken as early as in 2003. Li et al. [14] successfully joined two plates made of alloy Zr_45_Cu_48_Al_7_ using the laser welding method. Kim et al. [15] used an Nd:YAG pulsed laser when welding BMGs composed of Cu_54_Ni_6_Zr_22_Ti_18_. Kawahito et al. [16] successfully welded BMGs composed of Zr_55_Cu_30_Ni_5_Al_10_, using a precisely focused fiber laser beam. The tests involved the analysis of the course of crystallization without analyzing in detail how to maintain the amorphous nature of the fusion zone (FZ) and the heat affected zone (HAZ) during laser welding.

The reduction or even the entire elimination of crystallization during laser welding was presented by Wang et al. [17]. The researchers’ special attention was drawn to zirconium-based alloys because of their favorable mechanical properties, corrosion resistance, high GFA, and the extremely wide area of supercooled liquid [14,17,18,19]. To extend the range of the technical applicability of the aforesaid materials, it was necessary to perform tests [10,20] involving the joining of cast parts. The technologies applied in the tests enabled the obtainment of welds having the amorphous structure, yet with a very narrow range of welding parameters. The initial tests were concerned with the possibility of the welding of alloys from the Zr-Cu-Ni-Al system, performed using the Nd:YAG solid-state laser known to be characterized by the high density of welding energy and the possibility of obtaining adequate penetration depth. However, the zirconium-based alloys revealed a tendency to crystallize in the HAZ, thus leading to material brittleness, crack formation and, ultimately resulting in the deterioration of mechanical properties [17].

Crystallization occurring in the HAZ is the solid-state reaction differing from the crystalline phase formation taking place in the FZ [1,21]. Crystallization in the HAZ depends on heating and cooling times within the range of crystallization temperature during the welding thermal cycle. According to data in related reference publications [17], the making of the entirely amorphous weld requires the reduction of time at which the crystallization temperature affects specimens. An effective method enabling the reduction of the time of the effect of above-named temperature is the reduction of the initial welding temperature [22,23].

The technology enabling the joining of BMGs was selected through the analysis of reference publications. Because of the availability of necessary equipment and the possibility of the adjustment of related welding parameters, the selected method was that of laser welding performed using a TruLaser Station 5004 laser station.

### 1.2. The Influence of Varying Thermal Cycles During Welding on the Material Microstructure

The BMG joint made using the laser welding method consists of the FZ (weld), the HAZ, and the PM (Parent Material) [1,9]. After the use of adopted welding parameters, the structure and the properties of the PM usually remain unchanged. As a result, the quality of the welded joint depends entirely on the structure of the FZ and that of the HAZ. The fusion zone is the area of the melting of the material subjected to laser processing and followed by fast cooling, the rate of which depends on material and process parameters [9]. In the laser welding of alloys having the amorphous structure, the rate of cooling from the liquidus temperature (T_l_) to the glass transition temperature (T_g_) has a decisive influence on the fusion zone structure. The HAZ (i.e., the area adjacent to the FZ) is more susceptible to crystallization than the weld metal, as the heating time in the solid state is longer than the cooling time of the molten material [1]. In addition, the HAZ may undergo structural relaxation. The HAZ phase composition is unlikely to change because of the above-named structural relaxation, yet, the heat treatment at a temperature below the onset crystallization temperature (T_x_) may trigger changes of mechanical properties.

When adjusting laser welding parameters, the primary emphasis is given to the obtainment of desired weld morphology and penetration depth. Usually, the microstructure and the mechanical properties of the FZ and the HAZ are tested using X-ray-based methods, thermal analysis, calorimetric tests, electron microscopy, and hardness measurements [14,16,24,25,26]. Wang et al. in Reference [27] analyzed a change in the spot-weld structure in relation to laser beam parameters. As-cast plates were characterized by the presence of the amorphous-structure matrix and crystalline phase precipitates in the form of 5–25 µm-sized particles. In addition, tests performed using a transmission electron microscope revealed the presence of nanoparticles in the amorphous matrix. The use of adopted welding parameters resulted in the obtainment of a weld with full penetration. Because of the higher input power and significant laser beam power density characteristic of the Nd:YAG laser, the heating and cooling rates of the weld consisting of the amorphous-crystalline material were higher than the heating and cooling rates of the (solidifying) material in the as-cast state.

### 1.3. The Effect of Microstructure Changes on the Material Properties

The HAZ of Zr-based laser-welded BMGs tend to undergo crystallization [17,27]. The adopted laser beam parameters resulted in the formation of the significant amount of the Zr/Cu crystalline phase, leading to an increase in material brittleness, susceptibility to cracking in the above-named area, and a decrease in hardness. In addition, the HAZ crystallization affected the GFA of the BMGs subjected to welding. Laser welding parameters preliminarily adjusted in Reference [27] made it possible to restrain the precipitation of the Zr/Cu crystalline phase in the amorphous matrix. The division surface between the matrix and the microparticles contained few Zr/Cu phase precipitates and slight cracks. The X-ray tests and the EDS analysis of the crystalline phase revealed the presence of Cu and Zr, as well as the likelihood of the Zr_2_Cu phase precipitation. The priority of the Zr_2_Cu crystallization could be attributed to the fact that Cu and Zr were the primary compound constituents and that the Zr-Cu atomic pair was characterized by negative heat of mixing, the difference of atomic radiuses and electronegativity. In addition, it was observed that the Zr_2_Cu phase precipitated easier on the boundary between the amorphous-structure matrix and the reinforced particles.

## 2. Materials and Methods

The sample was a Zr_55_Cu_30_Ni_5_Al_10_ alloy. The procedure for obtaining BMGs in the form of plates involved: the production of an initial alloy in the form of an ingot, then casting the molten alloy into a copper mold. The tested ingot was prepared on the basis of pure Zr, Al, Cu, and Ni elements with a total weight of 30 g, using an induction generator in a ceramic crucible. The alloy composition represents nominal atomic percentages (Table 1).

The casting process of the liquid alloy into a copper mold involved: the melting of the initial alloy in a quartz crucible (using an induction generator), then raising the crucible plug, and introducing the molten alloy into the copper mold by the protective gas pressure. The investigated material was cast in form of plate with the dimension 20 × 10 × 2 mm.

Welding tests were performed with TruLaser Station 5004 device (Trumpf, Ditzingen, Germany). The parameters of the laser beam were selected on the basis of multiple tests. The TruLaser Station is a pulsed laser welding station. It enables positioning the working head by means of numerically controlled line guides. A stereoscopic microscope enables precise positioning of the head in the melting area and proper laser beam positioning on the welded material.

The device is integrated with a TruPulse 103 laser resonator of the average impulse power reaching 95 W and the maximum impulse power of 6 kW, as well as with a radiator enabling the exchange of resonator-generated heat with the environment. A laser beam generated in the resonator is transported to the working head via an optical fiber of a core diameter reaching 300 µm.

The parameters of the TruLaser Station used in this work are presented in the Table 2.

The laser beam parameters used in the process are presented in the Table 3.

**Figure 1:**
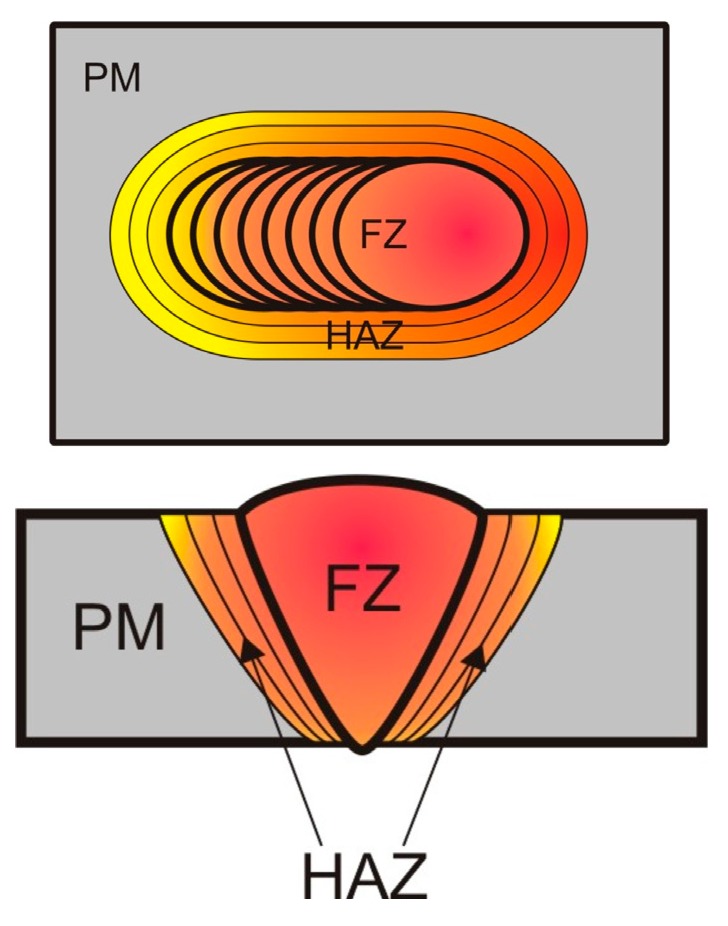
Scheme of the tested area.

The different weld zones are presented in Figure 1.

**Figure 2:**
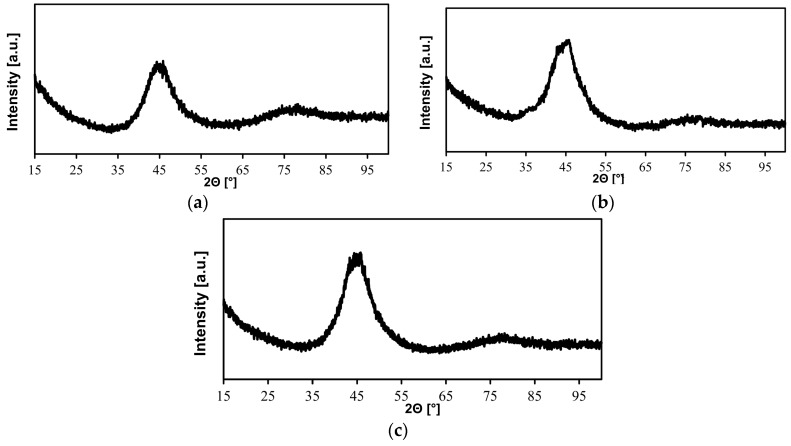
X-ray diffraction (XRD) patterns of the Zr_55_Cu_30_Ni_5_Al_10_ BMG in the form of a rod having a diameter of 2 mm surface of the rod (**a**); the cross-section (**b**); and in the form of a plate having a thickness of 2 mm (**c**) in the as-cast state.

The PANalytical XPERT PRO MPD X-Ray diffractometer (Philips, Surrey, UK) equipped with a copper anode X-ray tube was used to identify the phase composition of the HAZ and the FZ. The operating parameters of the lamp were 30 mA and 40 kV. For the purpose of the phase composition analysis, diffraction patterns were performed in the angular range 2Θ from 10° to 120°. Glassy structures were examined by X-ray diffraction (XRD) with the use of a Seifert—FPM XRD 7 diffractometer with Co Kα radiation at 35 kV. The data of diffraction lines were recorded by means of the stepwise method within the angular range of 30° to 90°. The diffraction pattern was obtained with step 0.04° 2Θ and 3 s per step. The X-ray wavelengths were applied at 1.79 Å (Figure 1) and 1.54 Å (Figure 2), respectively. The microscopic observation was executed by means of Titan 80–300 transmission electron microscope of FEI Company (Hillsboro, OR, USA). Nanomechanical properties tests were performed using the Hysitron TI 950 Triboindenter (Hysitron, Minneapolis, MN, USA) with the Berkovich indenter, equipped with QScope 250 microscopic attachment with Q-WM190 scanning probe.

## 3. Results and Discussion

### 3.1. Microstructure Test Results

Constituent elements of zirconium-based alloys are characterized by high affinity for oxygen, leading to greater amounts of impurities in alloys, easy formation of nuclei, and an increase in crystalline phase, ultimately resulting in the impairment of glass forming ability. The production of Zr-based BMGs is a complex process. Oxidation and crystallization may occur as early as when preparing a preliminary alloy, during casting, or even when preparing test specimens.

The analysis of X-ray spectra enabled the preliminary determination of specimen structures and the repeatability of results. The related test results revealed the good GFA of Zr-Cu-Ni-Al type alloys.

X-ray diffraction tests of the Zr_55_Cu_30_Ni_5_Al_10_ BMG (Figure 2) in the as-cast state revealed that the structure of the specimens subjected to analysis was amorphous.

The diffraction patterns of the 2-mm-thick test plates and rods having a diameter of 2 mm revealed wide diffuse spectra characteristic of the amorphous structure. The cross-sectional XRD pattern of the rod having a diameter of 2 mm revealed the presence of thin crystalline phase-related diffraction lines. The occurrence of weak diffraction reflections could imply the nucleation and the growth of crystalline phases during the fast cooling of metallic liquid.

The X-ray tests of alloys Zr_55_Cu_30_Ni_5_Al_10_ in the form of plates and rods enabled the preliminary verification of the structure, as well as the assessment of the GFA of the materials subjected to analysis and the ultimate selection of alloy Zr_55_Cu_30_Ni_5_Al_10_ for further tests.

**Figure 3:**
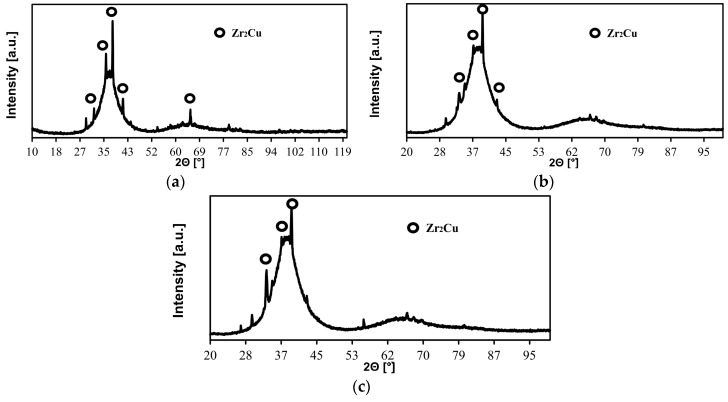
XRD pattern of the Zr_55_Cu_30_Ni_5_Al_10_ bulk metallic glass after laser welding process; area of PM of the plate close to the HAZ, the cross-section of the weld (**a**); P = 700 W, weld area, top view (**b**); and P = 1000 W, weld area, top view (**c**).

X-ray diffraction tests of Zr_55_Cu_30_Ni_5_Al_10_ BMGs after laser welding performed using a laser beam having a power of 700 W and 1000 W revealed that the structure of the above-named materials was amorphous-nanocrystalline. The XRD patterns of the PM and of the weld (Figure 3a) revealed wide and diffuse spectra characteristic of the amorphous structure and Zr_2_Cu crystalline phase-related thin diffraction lines.

The test results concerning the phase composition of the weld surfaces are also presented in Figure 3b,c). The diffraction tests of the Zr_55_Cu_30_Ni_5_Al_10_ BMG specimen exposed to the laser beam having a power of 700 W and 1000 W revealed the presence of amorphous phase-related broad, diffuse spectra as well as the presence of Zr_2_Cu crystalline phase-related low diffraction reflections.

The difference in the laser radiation power amounting to 300 W did not significantly affect the qualitative phase composition of the welds. The only changes involved a slight increase in the intensity of few Zr_2_Cu phase-related diffraction lines.

The X-ray diffraction tests of the Zr_55_Cu_30_Ni_5_Al_10_ BMG in the as-cast state and after welding revealed that the structure of the test materials was amorphous-nanocrystalline. The XRD pattern obtained in relation to the 2-mm-thick plate in the as-cast state revealed the presence of the amorphous phase-related line, whereas the XRD pattern concerning the plate in the post-weld state revealed the presence of diffraction reflections characteristic of the amorphous structure and crystalline phases. The X-ray test results revealed that nucleation are the most prevalent in the heat affected zone. The foregoing could be attributed to the chemical affinity of constituent elements (particularly Zr and Al) for oxygen, leading to the reduction of GFA, as well as to an excessively long time at which the HAZ was exposed to high temperature.

Transmission Electron Microscope (TEM) was used to verify the BMG structure and to analyze the nanocrystalline precipitates in the amorphous materials. The TEM-based test results did not provide complete information on the material structure. The tests involved the sampling of representative local fragments of specimens. It should be noted that the preparation of the specimens could be accompanied by crystallization or oxidation, affecting the structure of the materials subjected to microscopic observation.

**Figure 4:**
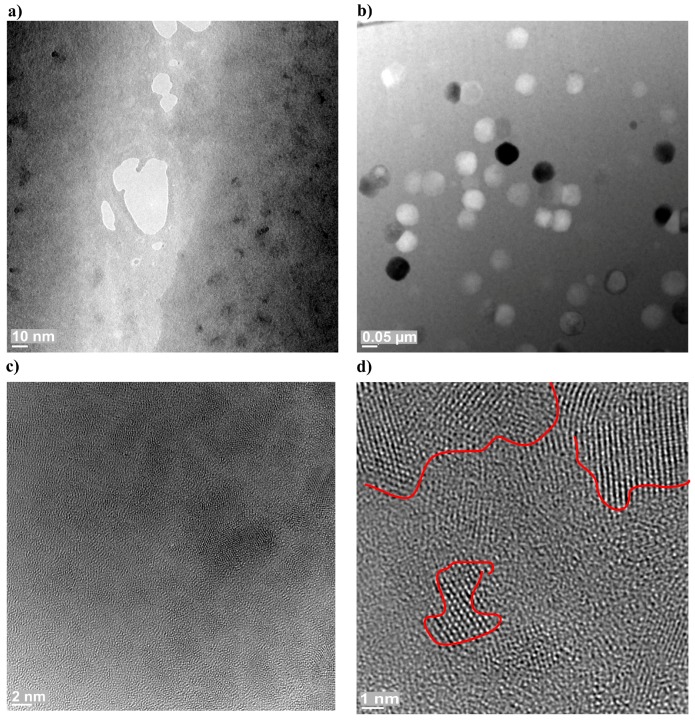
Amorphous-nanocrystalline structure image of the Zr_55_Cu_30_Ni_5_Al_10_ BMG in the form of plate having a thickness of 2 mm in the as-cast state (**a**,**b**) and the HRTEM images of the structure (**c**,**d**) of selected areas.

The identification and the analysis of the nanocrystalline features in the zirconium-based BMG and in the laser welds were performed using High Resolution Transmission Electron Microscope (HRTEM). Exemplary images of the amorphous-nanocrystalline structure, electron diffraction and the HRTEM image of the structure of selected areas of alloy Zr_55_Cu_30_Ni_5_Al_10_ in the form of 2-mm-thick plate in the as-cast state are presented in Figure 4.

**Figure 5:**
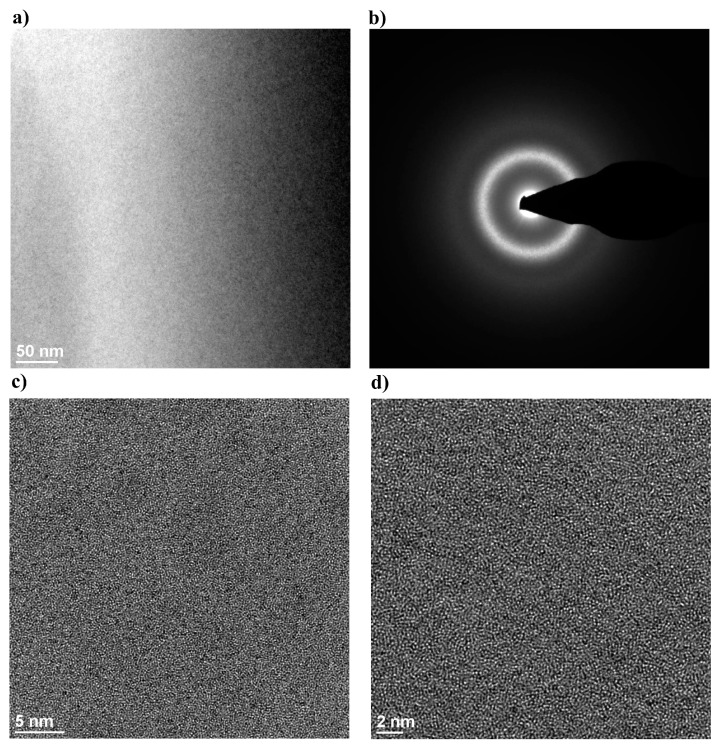
Image of the amorphous structure of the fusion zone in the weld made of the Zr-Cu-Ni-Al bulk metallic glass (**a**); electron diffraction pattern of the selected area (**b**); HRTEM-based structural images (**c**,**d**) of presented areas.

The microscopic test results revealed the presence of single, regular crystals in the amorphous phase. The size of the crystals was restricted within the range of 5 to 70 nm. The HRTEM-based observations of the FZ and HAZ structure in the weld made of Zr-based BMG were performed using alloy Zr_55_Cu_30_Ni_5_Al_10_ as an example. The observation in the bright field (Figure 5a) did not reveal the presence of regular crystals. Electron diffractions (Figure 5b) visible in related images were composed of diffuse spectra, which confirmed the amorphous structure of the FZ. In addition, Figure 5c,d presents the results of structural tests performed in a high-resolution mode. The high-resolution observations of the amorphous structure make it possible to perform detailed analyses of examined zones, yet they only represent selected fragments of the weld. The structural image along with the Fourier transform of selected FZ areas confirmed the lack of the regular arrangement of atoms in the test structure.

**Figure 6:**
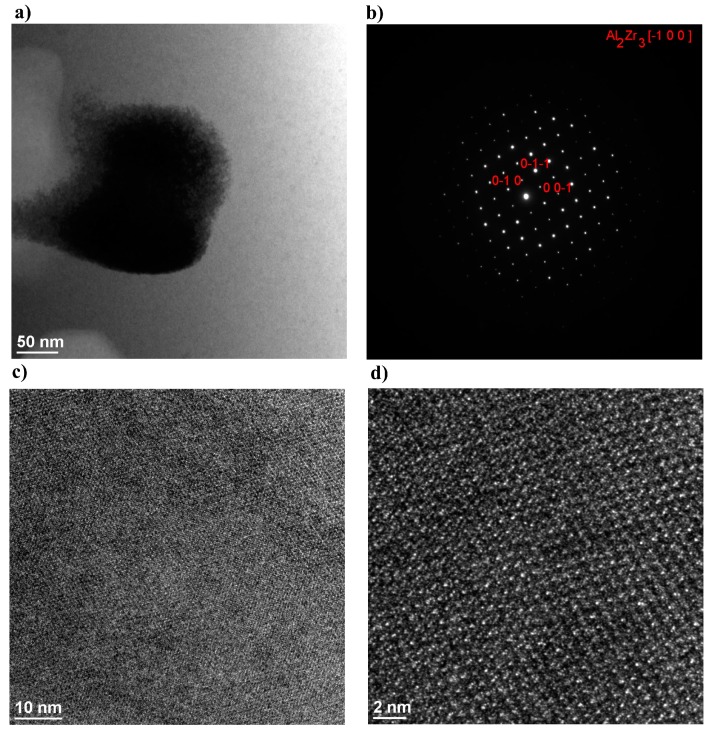
Image of the amorphous-crystalline structure in the heat affected zone of the weld made of the Zr-Cu-Ni-Al bulk metallic glass (**a**); electron diffraction pattern of the selected area (**b**); HRTEM-based structural images (**c**,**d**) of the presented area.

The structural observations of selected HAZ areas of the weld made of the Zr_55_Cu_30_Ni_5_Al_10_ BMG revealed the presence of single crystallites in the amorphous matrix. The structural images in the bright field revealed the presence of approximately 200 nm-sized crystallites (Figure 6a). The electron diffractions of selected crystalline precipitation areas (Figure 6b) confirmed the ordered structure of the test areas, whereas the electron diffractions of the amorphous matrix revealed the lack of long range order. The electron diffraction patterns revealed Al_2_Zr_3_ phase-related reflections. Figure 6c presents an additional image of the area between the crystalline phase and the amorphous matrix. The high-resolution image of the structure (Figure 6d) revealed the regular and periodic arrangement of atoms in the test structure. The tests revealed that the matrix was amorphous.

Observations related to the topography of welds made of Zr-Cu-Ni-Al BMG, performed using a Hysitron TI-950 Triboindenter equipped with a Q-Scope 250 probe, revealed the similar shape of the surface in individual zones of the weld. Because of the repeatability of test results, the work was provided with representative photographs presenting the surface of the welds made using laser welding.

**Figure 7:**
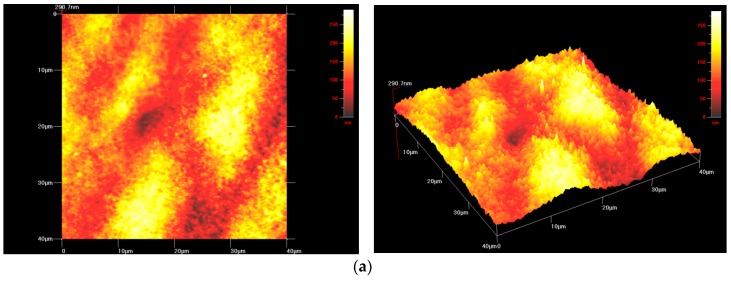

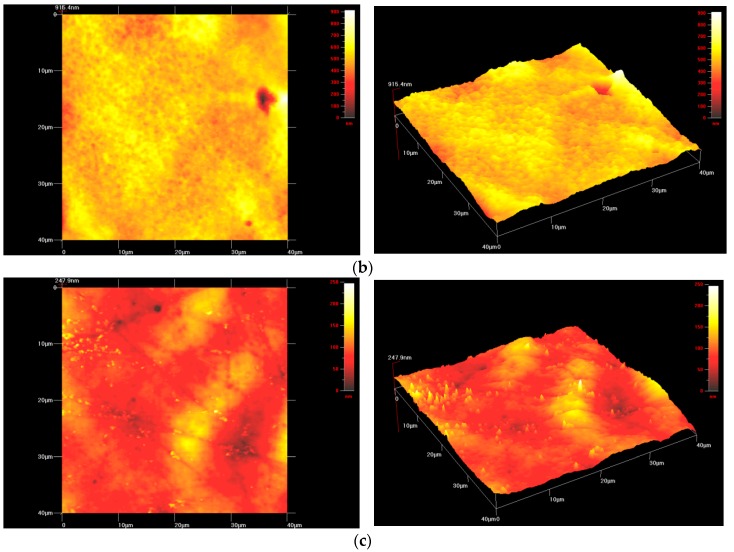
Surface topography in the area of the (**a**) parent material, (**b**) Zr-Cu-Ni-Al laser weld—parameters no. 1 and (**c**) heat affected zone—parameters no. 2 (2D image, 3D image; Atomic Force Microscope (AFM)).

The test results presented in 2D images were confirmed by surface morphology tests presented in the 3D space. The morphology of the PM, FZ, and HAZ surface is presented in Figure 7. The topography of the surface of the individual weld zones differed in the height of irregularities and roughness. Characteristic convexities presented in related examples might have resulted from the specific laser beam effect on the zirconium-based amorphous material and/or the non-homogenous chemical composition of the material subjected to welding.

The surface topography of the individual weld zones differed in height, sharpness, density of irregularities, and the degree of surface expansion. The presence of the amorphous-crystalline structure in the tested areas was confirmed by HRTEM-based examinations.

The visible surface morphology resulted from the direct laser beam effect on the Zr-based BMG followed by the fast cooling of molten metallic liquid. The weld made of the Zr_55_Cu_30_Ni_5_Al_10_ alloy contained few convexities in the FZ and HAZ. The tests concerning the FZ revealed slightly corrugated surface. The Zr-based alloys did not contain characteristic and clearly visible single convexities, frequently seen in the HAZ of Fe-based alloys [26].

### 3.2. Test Results Concerning Nanomechanical Properties

Tests of nanomechanical properties of selected welds made of Zr-based BMG involved nanohardness measurements and the determination of the Young’s modulus in the PM, FZ, and HAZ. The AFM and Triboindenter-based observations revealed the presence of convexities formed in defective surface areas (e.g., areas characterized by the non-homogenous chemical composition of the welded material resulting from the casting process or affected by the laser beam radiation). Other surface imperfections revealed in AFM-based images included nuclei of metallic phases or defects generated during mechanical surface preparation.

The literature date revealed that the correlation between properties and the structure was described using similar dependences regardless of materials, chemical compositions or technologies used when making specimens. The scientists [27], based on test results concerned with the correlation between the microstructure and the hardness in alloy Zr-Cu-Al-Ag-Ta-Si, revealed that the PM and the HAZ were characterized by similar hardness. The authors attributed the foregoing to the presence of very small crystallite or cracks in the above-named areas and ascribed the test result to the refinement degree and the number of micro-particles. Mechanical properties of materials composed of at least two phases are strictly related to the size distribution or the volumetric content of precipitated reinforcement [26]. It was ascertained that crystallization or precipitates in the amorphous matrix led to a significant increase in the material hardness. However, the tests discussed in the above-named research work revealed that the hardness in the HAZ was lower than that of the amorphous area. The scientists also emphasized that the lower value of hardness in the crystalline area could be ascribed to the presence of cracks near crystalline precipitates. Similar to tests [27], the hardness of the amorphous-structured weld did not differ significantly in the HAZ, FZ, and in the PM.

Measurement results concerning the reduced Young’s modulus and the nanohardness in the tested zones of the welds made of the Zr_55_Cu_30_Ni_5_Al_10_ BMG are presented in Table 4 and Table 5.

**Figure 8:**
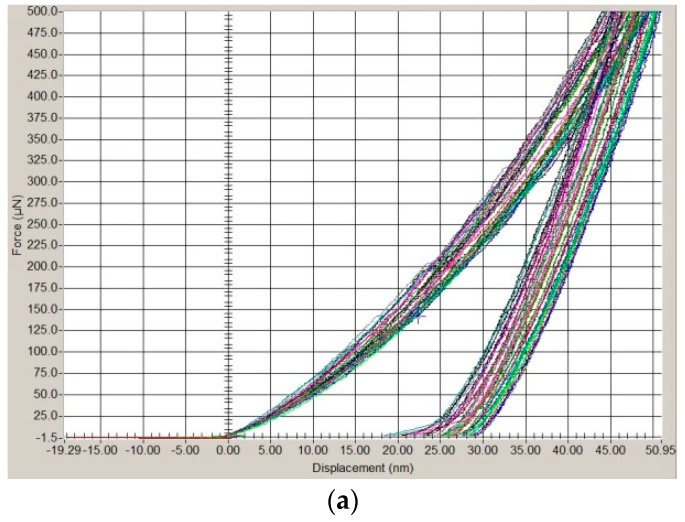

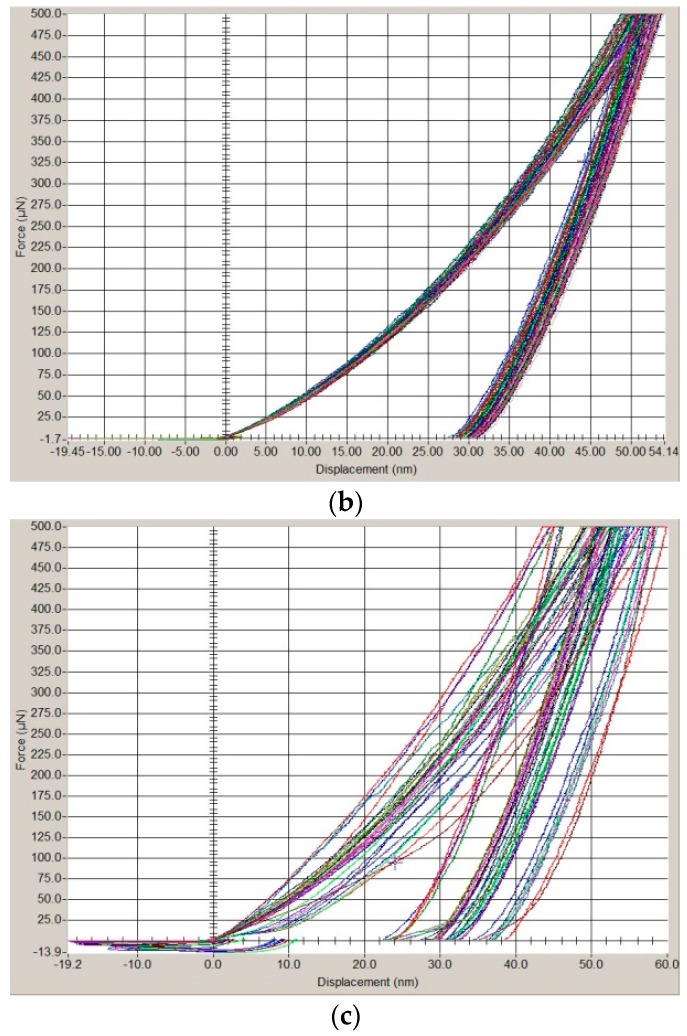
Displacement of indentation as the function of load in relation to the surface of the PM (**a**); FZ (**b**); HAZ (**c**) made of the Zr_55_Cu_30_Ni_5_Al_10_ alloy (Hysitron TI 950 Triboindenter, surface).

Curves determined in relation to the individual zones of the weld made of the Zr-based BMG, using present laser welding parameters no. 2 are presented in Figure 8.

Higher nanohardness and reduced Young’s modulus values were determined in relation to the laser weld made using an impulse energy of 2.78 J and an impulse peak power of 1000 W. H_v_ in the FZ and HAZ differed slightly, whereas E_r_ in the FZ and HAZ differed by 16.49 GPa in relation to the specimen made using laser beam parameters no. 1 and by 3.24 GPa in relation to the specimen made using laser beam parameters no. 2. Similar results related to the H_v_ measurement could imply the homogenous structure in the FZ and HAZ.

## 4. Conclusions

On the basis of research results analysis, the following conclusions have been voiced:

- The application of effective pressure die-casting into copper molds enabled the creation of bulk metallic glasses on zirconium matrix. The Zr_55_Cu_30_Ni_5_Al_10_ alloy in the form of 2-mm-thick plates and rods with 2 mm diameter were obtained.
- X-ray diffraction tests of Zr_55_Cu_30_Ni_5_Al_10_ BMGs after laser welding performed using a laser beam having a power of 700 W and 1000 W revealed that the structure was amorphous-nanocrystalline. The nucleation of crystalline phase Zr_2_Cu were the most prevalent in the HAZ.
- The microscopic test results of as-cast plates revealed the presence of single regular crystals in the amorphous phase. The size of the crystals was restricted within the range of 5 to 70 nm. The structural observations of selected HAZ areas of the weld revealed the presence of single approximately 200-nm-sized crystallites in the amorphous matrix, whereas HRTEM-based observations confirmed the amorphous structure of the FZ.
- The weld area of HAZ revealed slightly lower nanohardness than the nanohardness of smooth FZ and PM surface areas. E_r_ in the FZ and HAZ differed by 16.49 GPa in relation to the specimen made using laser beam parameters no. 1 and by 3.24 GPa in relation to the specimen made using laser beam parameters no. 2.

## Figures and Tables

**Table 1:** Chemical composition of Zr_55_Cu_30_Ni_5_Al_10_ alloy, purity, and shape of elements.

No.	Element	Mass (%)	At (%)	Purity (%)	Shape
1	Zr	67.01	55	99.9	lump
2	Cu	25.46	30	99.99	globule
3	Ni	3.92	5	99.9	pieces
4	Al	3.60	10	99.99	lump

**Table 2:** Laser station parameters.

Laser Station Parameters	
radiation wavelength	1064 nm
maximum average power	95 W
minimum impulse power	300 W
maximum impulse power	6 kW
minimum impulse time	0.3 ms
maximum impulse time	50 ms
maximum impulse energy	60 J
maximum repetition frequency	833 Hz
beam quality (BBP parameter)	12 mm × mrad
laser beam spot diameter	0.3–2.4 mm

**Table 3:** Laser beam welding parameters.

Laser Beam Welding Parameters	No. 1	No. 2
Peak power P (W)	700	1000
Duration time t (ms)	4	4
Impulse energy E (J)	2.08	2.78
Repetition frequency f (Hz)	4	4
Number of spots	15	15
Spot diameter d (mm)	0.3	0.3

**Table 4:** Measurement results concerning the reduced Young’s modulus and the nanohardness in the tested zones of the welds (SD—Std Dev).

Laser Beam Welding Parameters No. 1	E_r_ (GPa)	H_v_ (GPa)
Parent Materials	122.68 (±11.43 SD)	8.28 (±0.62 SD)
Fusion Zone	101.89 (±5.29 SD)	6.99 (±0.54 SD)
Heat Affected Zone	118.38 (±16.16 SD)	6.76 (±1.34 SD)

**Table 5:** Measurement results concerning the reduced Young’s modulus and the nanohardness in the tested zones of the welds (SD—Std Dev).

Laser Beam Welding Parameters No. 2	E_r_ (GPa)	H_v_ (GPa)
Parent Materials	118.20 (±6.97 SD)	8.55 (±0.56 SD)
Fusion Zone	106.92 (±4.21 SD)	7.38 (±0.25 SD)
Heat Affected Zone	110.16 (±8.94 SD)	7.13 (±1.05 SD)
